# Transcriptome analysis reveals differentially expressed lncRNAs between oral squamous cell carcinoma and healthy oral mucosa

**DOI:** 10.18632/oncotarget.16358

**Published:** 2017-03-18

**Authors:** Lu Feng, John R. Houck, Pawadee Lohavanichbutr, Chu Chen

**Affiliations:** ^1^ Program in Epidemiology, Division of Public Health Sciences, Fred Hutchinson Cancer Research Center, Seattle, Washington, USA; ^2^ Department of Head and Neck Surgery, The Affiliated Cancer Hospital of Zhengzhou University, Zhengzhou, Henan, P.R.China; ^3^ Department of Epidemiology, University of Washington, Seattle, Washington, USA; ^4^ Department of Otolaryngology, Head and Neck Surgery, University of Washington, Seattle, Washington, USA

**Keywords:** oral cavity and oropharyngeal squamous cell carcinoma (OSCC), long non-coding RNA, GEO, microarray

## Abstract

Oral cavity and oropharyngeal squamous cell carcinoma (OSCC) is a major cancer type in the head and neck region. To better understand the roles long non-coding RNA (lncRNA) play in OSCC carcinogenesis, we compared the expression levels of 3,054 probe sets for lncRNAs between 167 OSCCs and 45 healthy oral mucosa using an Affymetrix HG U133 plus 2.0 array dataset. We found 658 lncRNA transcripts (790 probe sets) to be significantly differentially expressed using a criteria of FDR < 0.01, with 36 of them (39 probe sets) showing more than a 2-fold change. We further validated the top differentially expressed lncRNAs in three independent datasets from Gene Expression Omnibus (GEO) repository: GSE42743, GSE9844, and GSE6791. Fourteen lncRNAs (15 probe sets) were validated in all three datasets using the criteria FDR < 0.01: *LOC441178, C5orf66-AS1, HCG22, FLG-AS1, CCL14/CCL15-CCL14, LOC100506990, TRIP10, PCBP1-AS1, LINC01315, LINC00478, COX10-AS1/LOC100506974, MLLT4-AS1, MIR31HG*, and *DUXAP10/LINC01296*. Three lncRNAs in the validated list which showed the highest fold change *(LOC441178, HCG22* and *C5orf66-AS1)* were verified by quantitative RT-PCR in a subset of 20 OSCCs and 10 control samples. In silico prediction of their functional role has given us directions for further investigation.

## INTRODUCTION

Oral cavity and oropharyngeal squamous cell carcinoma (OSCC) is the eighth most common cancer among men and fourteenth among women in the U.S. according to recent data [1]. The estimated incidence is 11.1 [SEER, http://seer.cancer.gov/] per 100,000 in the U.S. About 600,000 new cases arise annually worldwide [1–3]. Tobacco and alcohol consumption, as well as infection with high risk human papillomavirus (HPV), have been shown to be the main risk factors of OSCC in the U.S. [2, 4–6]. However, the precise mechanisms of OSCC carcinogenesis are not well understood; and little improvement has been made to the overall prognosis for advanced-stage OSCC in the past two to three decades, leaving the patients and their families with heavy disease burden [7]. There continues to be an urgent need to achieve a better understanding of the mechanisms of oral carcinogenesis in order to aid the discovery of effective therapeutic targets.

It is estimated that >70% of the human genome can be transcribed. However, only 2% are protein-coding and the majority of transcripts are not [8]. These majority transcripts are categorized as non-coding RNA (ncRNA). Except for those “housekeeping” ncRNAs such as tRNAs, rRNAs, there are also other mRNA-like transcripts which can be subdivided by length: small ncRNA (< 200 nt) and long ncRNA (> 200 nt) [8]. Small ncRNAs, like microRNAs, have been studied extensively and there is evidence to suggest that they may play an important role in cancer, including OSCC [9]. However, the study of lncRNAs in cancer has only begun in the last decade [10].

The functions of only a handful of lncRNAs have been studied with results showing that they play a role as transcriptional and post-transcriptional regulators. Their reported functions include: 1) Remodeling chromatin state (e.g. *HOTAIR* or *Xist* influences chromatin remodeling by recruiting PRC2 [11–13]); 2) Providing stability to proteins or protein complexes (e.g. *MALAT1* and *NEAT1* serve as molecular scaffolds for proteins within nuclear speckles and paraspeckles [10, 14]); 3) Competing with endogenous RNAs to modulate their functions ( e.g. *UCA1* and *MEG3* regulate oncogenes by “sponging” miRNAs and decreasing their function [15, 16]).

Gibb et al. was the first to report the expression of lncRNA in oral mucosa, implying lncRNAs contribute to the oral transcriptome [17]. Subsequent studies have evaluated whether lncRNAs are involved in tumor development by comparing their expressions between cancers and controls. Several such studies reported aberrantly expressed lncRNAs in oral cancers. *HOTAIR* (HOX transcript antisense RNA) was reported to be up-regulated in OSCC [18, 19] and laryngeal squamous cell carcinoma [20, 21]. An increase in its expression was associated with metastasis and poor prognosis of OSCC [18]. In 2015, Sharma S. et al. reported HPV16 oncoprotein E7 could be involved in cervical cancer carcinogenesis through regulating *HOTAIR* expression and function [22]. Whether this molecular event also occurs in HPV-related oral cancer remains unknown. *MALAT1* (metastasis associated lung adenocarcinoma transcript 1) is another reported lncRNA. This large, infrequently spliced non-coding RNA was aberrantly expressed in lung cancer and cervical cancer, and its upregulation is associated with growth and metastasis of several types of cancer, such as colorectal, pancreatic and gastric cancers [23, 24] and in tongue squamous cell carcinoma and OSCC [23, 25]. Other lncRNAs that have been reported in oral cancer include *MEG3*, which was down-regulated in tongue cancer [26], and *UCA1*, which was up-regulated in tongue cancer [27]. Some studies of head and neck cancer [20, 28–30] suggested that lncRNA, including *AFAP1-AS1, AB209630, GAS5*, and *HOTAIR*, could potentially serve as predictors of patient outcome or treatment response.

To better understand the role of lncRNAs in OSCC carcinogenesis, and to gain insight for the identification of potentially clinically relevant targets, we used a whole genome approach to examine expression differences of lncRNAs between OSCC and normal oral tissue. Previous studies by Risueño et al. [31], Liao et al. [32] and Michelhaugh et al. [33] have shown that existing microarray data can be mined to study lncRNA transcription. In this study, we developed our own approach to identify and validate a list of significantly dysregulated lncRNAs between OSCC and oral mucosa from healthy individuals using our previously generated microarray data.

## RESULTS

### Differentially expressed lncRNA genes between cases and controls

To identify differentially expressed lncRNAs between OSCC and control, we used a dataset comprised of 167 OSCCs and 45 oral mucosa samples from healthy controls previously generated using Affymetrix Human Genome U133 Plus 2.0 array (Affymetrix, Santa Clara, CA, USA). Out of 3,054 candidate probe sets that represent 2,172 lncRNA transcripts (see Supplementary Table 1), we found 790 probe sets (representing 658 lncRNAs transcripts) to be significantly differentially expressed with the criteria FDR < 0.01(see Supplementary Table 2). Of them, 568 were down-regulated and 222 were up-regulated in cancer. Amongst all, 39 probe sets (36 lncRNA transcripts) have a fold change difference > 2, with the majority (31 out of 39) of them being down-regulated. The two most differentially expressed probe sets identified the same lncRNA *LOC441178* with both showing a greater than 14-fold down-regulation in OSCC compared to control oral mucosa. To validate our findings in external datasets, we analyzed the expression of the top 39 probe sets that were identified in our study in three external datasets downloaded from GEO, adjusting for age and sex. Study participants’ characteristics for our study and for these three validation sets are summarized in Table 1. With a criteria of FDR < 0.01, most of the 39 probes were differentially expressed between cases and controls in at least one of the three GEO datasets. The 15 probe sets (representing 14 lncRNA transcripts) that were differentially expressed in all three validation datasets were as follows: *LOC441178, C5orf66-AS1, HCG22, FLG-AS1, CCL14/CCL15-CCL14, LOC100506990, TRIP10, PCBP1-AS1, LINC01315, LINC00478, COX10-AS1/LOC100506974, MLLT4-AS1, MIR31HG*, and *DUXAP10/LINC01296*. The results on their differential expressions are shown in Table 2.

**Table 1:** Characteristics of four microarray datasets used in this study

Characteristics		FHCRC dataset [34] (*n* = 212)	GSE42743 [35] (*n* = 103)	GSE9844 [36] (*n* = 38)	GSE6791 [37] (*n* = 49)
Case	Cancer	167 (OSCC)	74 (oral cavity)	26 (tongue)	35 (OSCC)
	Control	45 (healthy normal)	29 (adjacent normal)	12 (adjacent normal)	14 (non-cancerous normal)
Gender	Male	152	79	29	29
	Female	60	24	9	20
Age	< 50	64	29	11	7
	50~70	118	51	24	31
	> 70	30	23	3	11

**Table 2:** Validation of 39 differentially expressed probe sets between OSCC and control in three independent datasets

Probe sets ID	Gene Symbol	Chromosomal Location	Fold change in FHCRC dataset	*Q*-value in FHCRC dataset	*Q*-value in GSE42743	*Q*-value in GSE9844	*Q*-value in GSE6791
1563894_at*	***LOC441178***	chr6q27	–14.50	8.83E-49	1.00E-12	1.11E-06	6.09E-13
1561685_a_at*	***LOC441178***	chr6q27	–14.23	8.83E-49	5.22E-13	1.11E-06	2.26E-13
1558920_at	*SLC8A1-AS1*	chr2p22.1	–8.43	5.85E-45	6.51E-07	0.02	1.83E-03
236444_x_at	***C5orf66-AS1***	chr5q31.1	–6.60	1.91E-28	4.32E-09	2.29E-03	9.62E-06
227725_at*	*ST6GALNAC1*	chr17q25.1	–5.87	1.44E-15	1.93E-09	0.02	1.18E-04
204919_at*	*PRH1-PRR4/PRR4*	chr12p/chr12p13	–5.31	4.18E-06	5.29E-03	0.59	0.03
1560767_at*	***HCG22***	chr6p21.33	–3.95	1.91E-28	5.83E-14	2.35E-05	1.50E-05
241014_at	***FLG-AS1***	chr1q21.3	–3.61	6.52E-35	5.22E-13	6.69E-06	9.62E-06
244620_at	*SLC8A1-AS1*	chr2p22.1	–3.29	1.81E-34	4.33E-06	0.04	2.14E-03
224997_x_at	*H19/MIR675*	chr11p15.5	–3.23	6.43E-04	0.05	0.10	0.11
205392_s_at*	***CCL14/CCL15-CCL14***	chr17q11.2/chr17q12	–2.92	2.08E-14	1.29E-08	7.54E-06	7.39E-07
227917_at	***LOC100506990***	chr8p23.1	–2.77	4.44E-21	1.93E-09	1.04E-03	6.38E-04
242546_at	***DUXAP10/LINC01296***	chr14q11.2	2.75	3.56E-09	1.49E-06	8.29E-03	3.18E-04
202734_at*	***TRIP10***	chr19p13.3	–2.69	4.82E-33	3.06E-06	7.44E-03	6.29E-06
1557389_at	*SH3PXD2A-AS1*	chr10q24.33	–2.51	6.31E-14	2.99E-03	0.46	1.03E-03
1554097_a_at	***MIR31HG***	chr9p21.3	2.50	1.92E-09	1.48E-08	5.59E-03	8.48E-03
1562921_at	*EP300-AS1*	chr22q13.2	–2.44	3.79E-14	4.87E-03	0.04	3.30E-03
228658_at	*MIAT*	chr22q12.1	2.37	9.59E-09	2.31E-04	0.59	0.19
236573_at	*MIR1-2/MIR133A1/MIR133A1HG*	chr18q11.2	–2.34	2.46E-08	5.62E-07	0.17	0.12
227969_at	***PCBP1-AS1***	chr2p14	–2.31	1.24E-35	6.61E-08	5.41E-06	2.18E-04
208908_s_at*	*CAST*	chr5q15	–2.26	2.93E-30	7.59E-06	0.02	1.33E-03
229930_at	***LINC01315***	chr22q13.2	–2.25	1.77E-22	6.81E-09	2.35E-05	7.86E-06
239999_at	***LINC00478***	chr21q21.1	–2.25	2.34E-11	1.93E-09	2.35E-05	1.96E-06
220918_at	*RUNX1-IT1*	chr21q22.12	2.23	4.70E-11	3.42E-07	0.10	0.03
202672_s_at*	*ATF3*	chr1q32.3	2.23	9.56E-06	8.20E-05	0.31	0.03
1559361_at	*LOC101927668/MACC1*	chr7p21.1	–2.21	1.75E-13	7.55E-04	0.23	0.01
230451_at	***COX10-AS1/LOC100506974***	chr17p12	–2.20	2.23E-37	6.63E-09	1.11E-06	5.16E-08
219871_at	*KLF3-AS1*	chr4p14	–2.20	7.82E-19	5.00E-08	0.06	0.04
238320_at	*MIR612/NEAT1*	chr11q13.1	2.15	2.35E-06	0.02	0.29	0.02
207467_x_at*	*CAST*	chr5q15	–2.14	1.22E-30	3.68E-06	0.01	1.33E-03
1557146_a_at	*SSTR5-AS1*	chr16p13.3	–2.12	7.86E-07	0.02	0.40	0.19
210409_at*	***MLLT4-AS1***	chr6q27	–2.12	1.63E-30	6.45E-07	1.92E-04	2.38E-10
226382_at*	*LOC283070*	chr10p13	–2.09	9.82E-14	8.52E-06	9.58E-03	0.03
228370_at	*IPW/LOC101930404/PWARSN/SNORD107/SNORD115-13/SNORD115-26/SNORD115-7/SNORD116-22/SNORD116-28/SNORD116-4*	chr15q11.2	–2.09	9.34E-08	1.55E-06	0.02	0.04
232034_at	*LINC00537*	chr9q21.11	–2.05	1.33E-11	0.02	0.10	0.10
233565_s_at*	*FKBP1A-SDCBP2/SDCBP2*	chr20p13	–2.05	1.23E-09	4.78E-04	0.17	0.06
229635_at	*LINC01094*	chr4q21.21	2.04	4.78E-12	9.33E-05	0.05	0.01
228564_at	*LINC01116*	chr2q31.1	2.03	3.40E-10	4.94E-08	0.10	0.03
227061_at	*LINC01279*	chr3q13.2	–2.02	1.63E-03	5.34E-06	2.29E-03	0.19

Gene symbols in bold font refer to those that were validated in all three external datasets. * Probe sets that map to both coding and non-coding transcripts.

### Verification of *LOC441178*, *HCG22* and *C5orf66-AS1* by quantitative RT-PCR

**Figure 1:**
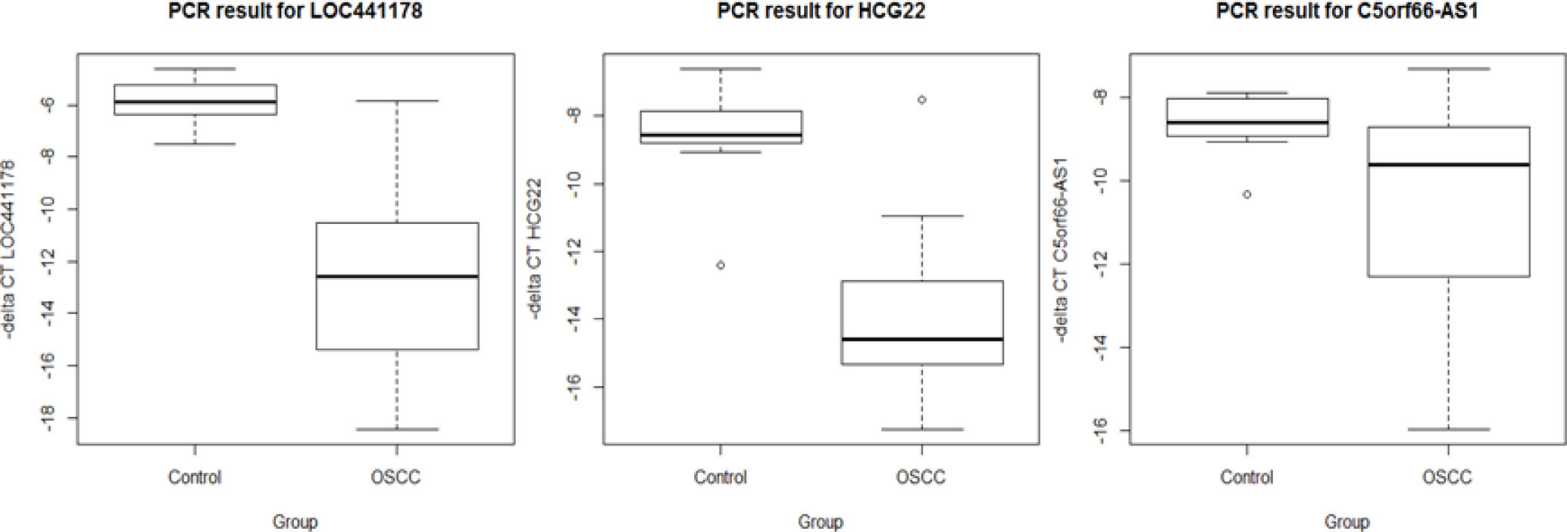
Expression level between OSCC and controls by qRT-PCR Results shown as delta Ct value standardized to beta actin. Delta Ct value in Y axis was transformed by multiplying -1 so that the higher value was corresponding to the higher expression level.

To confirm the differential expression signals found in the array data, three lncRNAs (*LOC441178, HCG22 and C5orf66-AS1*) in the validated list showing the highest fold changes were chosen to undergo qRT-PCR testing in a subset of 20 OSCCs and 10 normal controls samples (see Supplementary Table 3). The samples were randomly chosen from the original FHCRC study samples which consist 167 OSCCs and 45 oral mucosa from healthy people. *LOC441178* has two identified transcripts, one shorter transcript that codes for a 93 aa protein, and the other longer transcript is identified as a lncRNA. To confirm that the lncRNA transcript is truly differentially expressed, we designed a PCR primer pair that targets only the lncRNA transcript. Figure 1 shows the differential expressions of the three lncRNAs in OSCC and controls (*P* < 0.05). The correlation of Affymetrix array data and PCR results were calculated. Pearson's correlation coefficient value of *LOC441178, HCG22* and *C5orf66-AS1* were 0.88, 0.83 and 0.72, respectively (see Supplementary Figure 1).

### Prediction of putative targets of lncRNA *LOC441178* and functional clustering

Terai G et al. [38] have reported a comprehensive interaction database of lncRNA and mRNA based on sequence complementarity. We used that database to predict the targets of the No. 1 differentially expressed lncRNA *LOC441178*. The top 100 putative targets are shown in Supplementary Table 4. Functional annotation of these putative 100 target genes using DAVID bioinformatics classification tool [39] showed that they are enriched in 8 clusters with suggested functional involvement in cellular motility, ion channel signaling and ATP metabolism (see Table 3).

**Table 3:** Functional clustering of the putative predicted targets of LOC441178

Annotation Cluster 1Enrichment Score: 4.07393095028667				
Category	Term	Count	%	*P* Value
GOTERM_MF_DIRECT	GO:0016887~ATPase activity	11	11.22449	1.91E-08
GOTERM_MF_DIRECT	GO:0003777~microtubule motor activity	7	7.142857	2.46E-06
GOTERM_CC_DIRECT	GO:0030286~dynein complex	5	5.102041	3.80E-06
GOTERM_BP_DIRECT	GO:0007018~microtubule-based movement	6	6.122449	5.59E-05
GOTERM_CC_DIRECT	GO:0005874~microtubule	8	8.163265	8.78E-04
GOTERM_MF_DIRECT	GO:0005524~ATP binding	17	17.34694	0.002009
KEGG_PATHWAY	hsa05016:Huntington's disease	6	6.122449	0.003745
GOTERM_CC_DIRECT	GO:0005929~cilium	4	4.081633	0.038842

Annotation Cluster 2Enrichment Score: 3.17701545165616				
Category	Term	Count	%	*P* Value
GOTERM_CC_DIRECT	GO:0042383~sarcolemma	7	7.142857	3.92E-06
GOTERM_BP_DIRECT	GO:0010881~regulation of cardiac muscle contraction by regulation of the release of sequestered calcium ion	4	4.081633	1.14E-04
GOTERM_BP_DIRECT	GO:0010882~regulation of cardiac muscle contraction by calcium ion signaling	3	3.061224	5.23E-04
GOTERM_BP_DIRECT	GO:0002027~regulation of heart rate	4	4.081633	6.12E-04
GOTERM_CC_DIRECT	GO:0030315~T-tubule	4	4.081633	8.67E-04
GOTERM_BP_DIRECT	GO:0060048~cardiac muscle contraction	4	4.081633	0.001524
GOTERM_MF_DIRECT	GO:0044325~ion channel binding	5	5.102041	0.002229
GOTERM_BP_DIRECT	GO:0035994~response to muscle stretch	3	3.061224	0.002902
GOTERM_CC_DIRECT	GO:0014704~intercalated disc	3	3.061224	0.020809

Annotation Cluster 3Enrichment Score: 2.9557718510137274				
Category	Term	Count	%	*P* Value
GOTERM_BP_DIRECT	GO:0045053~protein retention in Golgi apparatus	3	3.061224	2.51E-04
GOTERM_BP_DIRECT	GO:0006623~protein targeting to vacuole	3	3.061224	6.95E-04
GOTERM_CC_DIRECT	GO:0019898~extrinsic component of membrane	4	4.081633	0.007787

Annotation Cluster 4Enrichment Score: 2.830552043347304				
Category	Term	Count	%	*P* Value
GOTERM_CC_DIRECT	GO:0042383~sarcolemma	7	7.142857	3.92E-06
GOTERM_CC_DIRECT	GO:0043034~costamere	4	4.081633	1.07E-04
GOTERM_CC_DIRECT	GO:0045211~postsynaptic membrane	7	7.142857	6.23E-04
GOTERM_MF_DIRECT	GO:0005200~structural constituent of cytoskeleton	3	3.061224	0.099947
GOTERM_CC_DIRECT	GO:0045121~membrane raft	3	3.061224	0.270657

Annotation Cluster 5Enrichment Score: 1.6982330480366516				
Category	Term	Count	%	*P* Value
GOTERM_BP_DIRECT	GO:0060048~cardiac muscle contraction	4	4.081633	0.001524
KEGG_PATHWAY	hsa05410:Hypertrophic cardiomyopathy (HCM)	3	3.061224	0.068115
KEGG_PATHWAY	hsa05414:Dilated cardiomyopathy	3	3.061224	0.077481

Annotation Cluster 6Enrichment Score: 1.1092710148648803				
Category	Term	Count	%	*P* Value
GOTERM_MF_DIRECT	GO:0016874~ligase activity	6	6.122449	0.010124
GOTERM_MF_DIRECT	GO:0004842~ubiquitin-protein transferase activity	5	5.102041	0.076177
KEGG_PATHWAY	hsa04120:Ubiquitin mediated proteolysis	3	3.061224	0.173404
GOTERM_BP_DIRECT	GO:0016567~protein ubiquitination	4	4.081633	0.273313

Annotation Cluster 7Enrichment Score: 0.898891114842629				
Category	Term	Count	%	*P* Value
GOTERM_MF_DIRECT	GO:0005089~Rho guanyl-nucleotide exchange factor activity	3	3.061224	0.053963
GOTERM_BP_DIRECT	GO:0035023~regulation of Rho protein signal transduction	3	3.061224	0.063457
GOTERM_MF_DIRECT	GO:0005085~guanyl-nucleotide exchange factor activity	3	3.061224	0.112325
GOTERM_BP_DIRECT	GO:0051056~regulation of small GTPase mediated signal transduction	3	3.061224	0.147561
GOTERM_BP_DIRECT	GO:0043547~positive regulation of GTPase activity	6	6.122449	0.158154
GOTERM_BP_DIRECT	GO:0043065~positive regulation of apoptotic process	3	3.061224	0.450355

Annotation Cluster 8Enrichment Score: 0.8894138004429241				
Category	Term	Count	%	*P* Value
GOTERM_MF_DIRECT	GO:0004672~protein kinase activity	5	5.102041	0.09728
GOTERM_MF_DIRECT	GO:0004674~protein serine/threonine kinase activity	5	5.102041	0.11034
GOTERM_BP_DIRECT	GO:0006468~protein phosphorylation	5	5.102041	0.199986

## DISCUSSION

In this study, we identified 790 significantly differentially expressed probe sets, representing 658 lncRNAs, between OSCCs and normal oral mucosa, with 39 of them, representing 36 lncRNAs, showing more than a 2-fold change in their expression levels. We also used three independent datasets to validate our findings, and 14 lncRNAs were found to be significantly differentially expressed in all data sets. We suspect these differentially expressed lncRNAs in OSCC may be involved in oral cancer carcinogenesis, and they also could serve as new choices for the investigation of potential OSCC biomarkers and/or therapeutic targets. Some of our findings are novel in that of the 14 validated lncRNAs, more than a half (8 out of 14) have never been reported to be associated with cancer before: *LOC441178, COX10-AS1, PCBP1-AS1, FLG-AS1, MLLT4-AS1, LINC01315, LOC100506990*, and *CCL15-CCL14*. *LOC441178* was the top differentially expressed lncRNA in all four datasets, and was confirmed by qRT-PCR in a subset of samples. Prediction of targets of lncRNA *LOC441178* in silico using a lncRNA-RNA interactions database [38] showed that *LOC441178* will potentially interact with both coding and non-coding RNAs, some of which are related to cancer, such as coding mRNA *MUC16/CA125*[40], and non-coding RNA *KCNQ1OT1* [41]. Functional clustering of these predicted targets give us an initial glance at the biological functions associated with this top lncRNA. With more than 100 fold change difference between cancer and control in qRT-PCR testing, it may have strong potential as a diagnostic biomarker for OSCC. *HCG22* was found to be down-regulated in oral cancer and its lower expression was reported to be associated with poor survival in a recent lncRNA study using TCGA data [42]. While our qRT-PCR results confirm the down-regulation of *HCG22* in OSCC, we did not find an association between *HCG22* expression levels and survival in our study population (data not shown). *C5orf66-AS1*, also known as *Epist*, was previously found to be downregulated and may act as a tumor suppressor in esophageal squamous cell carcinoma [43]. It has the same expression pattern in OSCC (see Figure 1), suggesting that *C5orf66-AS1* may be involved in multiple cancer types that occur in the upper aerodigestive tract. Within 5kb distance of *C5orf66-AS1* is pituitary homeobox 1 (*PITX1*). *PITX1* expression level was found recently to be a novel predictor for treatment response of head and neck cancer [44]. Further study is needed to see if *C5orf66-AS1* could be a regulator of this neighboring *PITX1* gene. MIR31 host gene (*MIR31HG*), also named lncRNA *LOC554202*, was found to be down-regulated in gastric cancer [45] and bladder cancer [46], but up-regulated in breast cancer [47]. Further, there is evidence showing that it was upregulated in oncogene-induced senescence process; and knockdown of *MIR31HG* induced a tumor suppressor p16INK4A dependent senescence phenotype, which might link it to HPV-related cancer carcinogenesis [48]. In our study, *MIR31HG* expression does not appear to be significantly different between HPV-positive OPC (oropharyngeal cancer) and HPV-negative OPC (*P* = 0.02, lower in HPV-positive OPC). However, we found it was significantly differentially expressed between subsites of OSCC (OPC vs. OCC (oral cavity cancer), *P* = 9.29E-07, lower in OPC), and between smokers and non-smokers (*P* = 5.05E-05, lower in smokers). *LINC01296* was reported to be dysregulated in colorectal cancer and was suspected to be a potential prognostic biomarker [49]. The expression level of the probe set for *LINC01296* in our study was also significantly different between OSCC and controls, suggesting that this lncRNA may be involved in carcinogenesis process.

Some previously reported cancer-associated lncRNAs, such as *HOTAIR, UCA1*, and *MALAT1*, were found to be differentially expressed in our study, but none was in our top, validated list. Interestingly, some lncRNAs showed opposite expression direction in our study than in previous studies focusing on individual lncRNAs in head and neck cancers. Supplementary Table 5 shows their deregulation status in other studies and in our data. The discrepancies perhaps could be related to differences in sample size, platform used, origin of the control tissue used, or tumor site.

There are a large number of lncRNAs with unknown function that have yet to be studied. While genome wide study of lncRNA is a good approach to unveil more lncRNAs that may be involved in carcinogenesis or may represent potential therapeutic targets, making direct comparisons of results from various studies has been difficult. A major challenge is the use of different assay platforms by different studies. The lack of standardization in gene nomenclature is also an issue. Several studies of head and neck squamous cell carcinomas (HNSCC) have been carried out based on deep sequencing data from TCGA [42, 50, 51]. Two other studies have looked at lncRNA expression using re-annotated microarray data in OSCC. Even when the same microarray platform or a different version of the same microarray platform was used, difficulty in direct comparison remains. Gao et al. [52] found eight differentially expressed lncRNAs by comparing 26 tongue squamous cell carcinomas versus 12 controls, using the same Affymetrix U133 Plus 2.0 Gene Chip array data like we did. None of the eight appeared in our differential expression list. Differences in tumor site, sample size, and probe sets reannotation processing may be the reasons between their results and ours. Zhang et al. [53] compared 57 OSCCs and 22 normal controls using an Affymetrix Human Exon 1.0 Array. In their results, 160 lncRNAs were found to be dysregulated in cancer, of which seven were found in our 658 lncRNA list (*LOC645949, LINC00896, FAM99B, LINC00167, LINC01056, SND1-IT1, NCBP2-AS2*). The reasons of so few overlaps are unknown and could be related to different version of the Affymetrix platform used and the difference in data processing. Zhang et al. excluded probe sets that map to multiple transcripts in their analysis; while we did not. Excluding probe sets that also identify coding transcripts may mean a loss of potential lncRNA of interest. In our study, we chose to keep those probe sets in the initial analysis, because in some cases, it is possible to determine lncRNA transcript expression status more definitively through other methodologies such as quantitative RT-PCR. This approach allowed us to identify our most differentially expressed lncRNA *LOC441178*.

The recent development of using CRISPRi method for high throughput functional screening of lncRNAs has increased the number of lncRNAs with known functions [54]. However, this is beyond the scope of the current report. *In silico* prediction of the potential targets and the pathway analysis of these targets gave us some suggestion to the possible biological functions of our top differentially expressed lncRNA. However, further research is needed to explore more on these functions and the potential usage as biomarkers and/or therapeutic targets for OSCC.

## MATERIALS AND METHODS

### Gene expression array data and identification of lncRNAs

The gene expression microarray data used in this study was generated previously by our group as part of an OralChip study at the Fred Hutchinson Cancer Research Center (FHCRC). The study was designed to identify gene expression profiles that are related to clinical outcomes of OSCC [34, 35, 55, 56]. The microarray data were generated using the Affymetrix Human Genome U133 Plus 2.0 array (Affymetrix, Santa Clara, CA, USA) and were deposited in the Gene Expression Omnibus (GEO) Repository under the general accession number GSE30784. The dataset has 212 samples in total, 167 of them are OSCCs and 45 of them are oral mucosa from healthy controls. The samples and patients’ clinical data including age, gender, tumor site, stage, and HPV status were obtained from the original OralChip study. This investigation has been approved by the institutional review office of Fred Hutchinson Cancer Research Center. All microarray data have passed two rounds of quality control checks [34, 56]. CEL files were preprocessed and normalized using RMA algorithm in Partek Genomics Suite™ software version 6.6. To determine the candidate probe sets on the microarray that represent lncRNAs, first, we matched lncRNAs identified by HGNC (HUGO Gene Nomenclature Committee) Database [57] (downloaded from http://www.genenames.org/ at 1/27/2016) with Affymetrix probe ID using Entrez gene ID or Ensemble gene ID. 2,183 probe sets were matched to transcripts identified as “RNA, long non-coding” by HGNC; second, we identified 2,488 probe sets mapped to lncRNA from the Affymetrix annotation file (downloaded from http://www.affymetrix.com/ at 1/25/2016, netaffx-build=35) for the U133 2.0 Plus array. After removal of 1,617 duplicates, we obtained a final list of 3,054 probe sets that represent 2,172 lncRNA transcripts.

### Statistical analysis

The statistical analysis of differentially expressed lncRNA (between cases and controls) was performed using linear regression, adjusting for age and sex. The false discovery rate (FDR) of 0.01 and a 2-fold difference in expression between the two groups were used as criteria to select the differentially expressed lncRNA. All analyses were performed using R version 3.3.0.

### Validation using independent datasets

In order to validate our findings, we searched Gene Expression Omnibus (GEO) (http://www.ncbi.nlm.nih.gov/geo/) for datasets with OSCC and normal control samples that also used the Affymetrix U133 Plus 2.0 Gene Chip array. Three datasets were identified: GSE42743 with 74 oral cavity squamous cell carcinoma and 29 adjacent normal tissue [35]; GSE9844 with 26 tongue squamous cell carcinoma and 12 matched adjacent normal tissue [36]; and GSE6791 comprised of 28 cervical cancers, 42 head and neck cancers and 14 site-matched normal oral tissue [37]. We used these three data sets to validate our findings after excluding samples of irrelevant anatomic site and samples containing missing data. The false discovery rate (FDR) < 0.01 was used as criteria in each dataset during validation comparison.

### Quantitative reverse transcription polymerase chain reaction (qRT-PCR)

Among the validated list of lncRNAs, we chose three (*LOC441178, C5orf66-AS1 and HCG22*) with the highest fold change to do further validation by performing qRT-PCR using a subset of 20 oral cancer samples and 10 control samples randomly chosen from samples in the OralChip study. Each sample was assayed in triplicate in 10 uL reaction volumes using the QuantiTect SYBR Green RT-PCR kit (Qiagen, Valencia, CA, USA) on a 7900HT Sequence Detection System (Life Technologies, Carlsbad, CA, USA). The cycling conditions were as follows: 30 min incubation at 50°C, 15 min incubation at 95°C, and 40 cycles of 15 s at 94°C, 30 s at 55°C, and 30 s at 72°C. Ten-point standard curves were generated using Total RNA - Human Normal Tissue Tongue (Biochain Inst., Newark, CA, USA) for all three genes. The linear correlation coefficient (R2) was ≥ 0.99 for all runs. The mean threshold cycle (Ct) values were calculated from the triplicate Ct values. Samples that had Ct values with SD > 0.35 in their triplicate run were repeated. Mean Ct values were standardized to the mean Ct value of beta actin housekeeping gene (Quantitect primers (Qiagen, Valencia, CA, USA). Correlations between qRT-PCR and microarray data were performed using R Stats Package. Primer pairs used for each gene were as follows: LOC441178*: Forward primer* GGGTATTTTGTGC TCCCCCA; *Reverse primer* CAGGCACTGAAGGTTCG GAT; HCG22*: Forward primer* ACAGCAGTGAAACC CACCA; *Reverse primer* GAAGCCCAATCCAACA AAGAGC; C5orf66-AS1: *Forward primer* GCTTCGCGTC AAGAGGGTAT; *Reverse primer* AAGCCGCGGGAA TGTCTTTA. *P* value was calculated using student's t test between groups.

### Functional predictions

Prediction of the putative targets of lncRNA *LOC441178* was done using a comprehensive interaction database of lncRNA-mRNA which was reported by Terai G et al. [38] in 2016. GO ontology and KEGG pathway analysis and functional clustering of the top 100 targets was done by a gene functional classification tool, DAVID Bioinformatics Resources 6.8, NIAID/NIH [39]. The enrichment thresholds were by the web tool default and the enrichment score was calculated automatically by the tool.
